# To Raise the Standard of Medical Education

**Published:** 1894-04

**Authors:** 


TO RAISE THE STANDARD OF MEDICAL EDUCATION.—A meeting of the committee of the Association of the American Medical Colleges was recently held, and the following resolutions adopted, to present to the Association in June at San Francisco:

The committee agreed upon the following as necessary to admission to any of the seventy-one colleges forming the American Association:

English composition, in candidate's handwriting, not less than two hundred words, composition to include spelling, punctuation, paragraphing and construction. Examination in arithmetic, algebra through quadratics, and elementary physics. Latin to the extent of one year's study as indicated by Harkness's Latin Reader.

Graduates or matriculates of reputable colleges and high schools of the first grade, or normal schools established by State authority, or who have successfully passed the examination provided by the State of New York, may be exempt from the requirements named.

Students deficient in one or more branches named as requirements shall have time until the beginning of the second year to make up such deficiency; provided, however, that students who fail in any two of the requirements in this second examination shall not be admitted to the second course.

A resolution was adopted to recommend to the Association that after the year 1895 no medical college shall be permitted to remain or become a member of the Association that does not provide either for a three-year course of eight months' study or a four-year course of not less than six months in each year.

A sub-committee was ordered to be appointed to prepare a curriculum of studies in the medical colleges providing for the minimum of time and lectures to be devoted to each one. This sub-committee will report to the general committee at the San Francisco convention.

